# Perceived hope affects mental health among Portuguese Adults in year three of the COVID-19 Era: The mediation role of well-being

**DOI:** 10.1192/j.eurpsy.2025.536

**Published:** 2025-08-26

**Authors:** C. Laranjeira, A. Querido

**Affiliations:** 1 School of Health Sciences; 2 ciTechCare, Polytechnic Institute of Leiria, Leiria, Portugal

## Abstract

**Introduction:**

In recent years, more and more researchers have found positive psychological changes after experiencing stressful events. When people are exposed to stressful events, a sense of hope and psychological well-being, as significant positive psychological traits, can lessen the negative effects of psychological imbalance and help them cope with their worries in life in a more positive way, effectively promoting and protecting their mental health

**Objectives:**

This study aims to examine the mediating role of well-being in the relationship between people’s perception of hope and mental health.

**Methods:**

The present research was performed using a convenience (or snowball) sampling method obtained in the context of the Hope Barometer research program in 2023. Inclusion criteria were being an adult (aged ≥ 18 years); providing e-consent; having internet access; and understanding the Portuguese language. An online survey including information sheet, demographic questions, and three instruments, namely: Dispotional Hope was measured through the Perceived Hope Scale [PHS]; Mental health status was evaluated by General Anxiety Disorder-7itens; and, well-being measured by the Mental Health Continuum Short Form (MHC-SF). We employed the PROCESS macro for SPSS (model 4: mediation analysis) to evaluate our model.

**Results:**

The most frequently reported demographic categories were female (n=402), married (n = 206), have children (n = 344), graduated (n = 442), and with religious/spiritual affiliation (n = 400). The mean age was 47.72±11.86 years old. Dispositional hope were significantly and positively correlated with mental health. We conducted a mediation analysis to examine whether mediates the relationship between dispositional hope and mental health. The indirect effects for hedonic well-being (β = 0.28; 95% CI [0.02, 0.36]), psychological well-being (β = 0.14; 95% CI [0.06, 0.19]), and social well-being (β = 0.06; 95% CI [0.008, 0.073]) were all significant, indicating a mediating effect.

**Conclusions:**

Given the COVID-19 pandemic and its consequences have caused a variety of psychological distress such as fears, worries, and anxiety among people worldwide, this study underlies the mechanism between positive psychological resources such as perceived hope and well-being of individuals during the times of crisis of COVID-19 affects their mental health.

**Disclosure of Interest:**

None Declared

